# Gender Differences in Research Project Grants and R01 Grants at the National Institutes of Health

**DOI:** 10.7759/cureus.14930

**Published:** 2021-05-10

**Authors:** Amna Mohyud Din Chaudhary, Sadiq Naveed, Beenish Safdar, Sundas Saboor, Muhammad Zeshan, Faisal Khosa

**Affiliations:** 1 Psychiatry, Nishtar Medical College & Hospital, Multan, PAK; 2 Psychiatry, institute of Living, Hartford, USA; 3 Graduate Medical Education, John T. Mather Memorial Hospital, Northwell Health, Port Jefferson, USA; 4 Psychiatry, Khyber Medical College, Peshawar, PAK; 5 Psychiatry/Child and Infant Psychiatry, Rutgers Medical School, Newark, USA; 6 Psychiatry, Bronx Lebanon Hospital, Icahn School of Medicine at Mount Sinai, Bronx, USA; 7 Radiology, Vancouver General Hospital, Vancouver, CAN

**Keywords:** gender, gender disparity, nih funding gender disparity, female researcher, academic productivity, research career, r01 grant, r01-equivalent grant, nih funding

## Abstract

Objectives

The National Institutes of Health (NIH), which is the world's largest funding source for research, offers various types of competitive grants depending on the duration, research type, and budget. The Research Project Grant (RPG) is the oldest mechanism for grant allocation that is used by the NIH. In this study, we explored the gender trends of NIH RPGs and R01 grants over the last two decades.

Methods

By utilizing the NIH Research Portfolio Online Reporting Tool (RePORT), data for gender were extracted, and the percentage of women as RPGs Investigators, R01-equivalent grant including R01 type 1 and type 2 grant awardees, from 1998 to 2019 were tabulated. The absolute change was calculated.

Results

From 1998 to 2019, the percentage of female RPG awardees has increased. However, the success rates for female RPG applicants have decreased during the same period. The funding and success rates for new R01 awards have been similar for both men and women, but women have been less successful at the renewal of R01-equivalent awards.

Conclusion

Gender disparity exists in awardees of higher RPGs, including the R01 award. This highlights the need for further actions to ensure gender parity in grant allocations at the NIH.

## Introduction

Women have narrowed the gender gap with men among biomedical science degree holders, but they are lagging behind in academic ranks and leadership positions [1-3]. Women were 49% of all PhD degree awardees in biological sciences in 2005, and it increased to 52% in 2010 [4]. However, female assistant professors with a biology PhD constituted only 44% of the faculty in 2015 [5]. Similar discrepancies between PhD holders and faculty representation also persist in academic medicine [2,6,7], professional medical societies [8-10], and editorial boards of medical journals [11,12]. The National Institutes of Health (NIH), which is the world’s leading source of public funding for biomedical research, receives disproportionally a lower number of newer grant applications from women [13]. Furthermore, less than one-third of NIH research grantees are comprised of women, even though their success rates are similar for obtaining first grants compared to men [14].

The NIH achieves its goal of expanding biomedical research by supporting research in different domains through the administration of various grant types [15]. These grants are pivotal for scientists to carry out research activities, run their laboratories, and apply for promotions and tenure [16]. The NIH also plays a pivotal role in the contribution to the nation’s economy by creating jobs and increasing the demand for local services as well as serve as a foundation for the United States (U.S.) biomedical industry. It is estimated that approximately every US dollar of NIH funding generates ≈$2.21 in local economic growth [17].

Various funding opportunities are available through the NIH. The Research Project Grant (RPG) is the original and oldest mechanism of grant used by the NIH. The NIH grants are specified by an activity code representing the type of research being funded [13]. The R01 award is the “gold standard” of research awards and provides funding and support for health-related research and development based on the mission of the NIH [17]. The NIH has two types of applications for R01 grants including R01 New (Type 1) and R01 Renewal (Type 2) [18].

It is critical to explore the trends of gender disparity in NIH funding to address academic and scholarly diversity. In our study, we explored the trends of the NIH R01 grant and characterized the potential explanations for any existing gender differences. We also analyzed NIH funding trajectories over time, comparing gender differences for the early stage of women's careers and whether they continue to stay funded at the same rates as men. We explored the differences in funding trajectories for men and women from 1998 to 2019, using NIH grant records for investigators who received a major NIH RPG for the last two decades.

## Materials and methods

Our methodology has been validated in recent publications [19]. This retrospective study did not require Institutional Ethics Board approval as the data were exported from publicly available data at the NIH Research Portfolio Online Reporting Tools (RePORT) - NIH Data Book. We utilized the NIH grant and funding reports for the consecutive fiscal years 1998 to 2019.

Variables

The percentage of women receiving RPGs, and the differences in the annual applications, awards, and success rates for the RPGs by gender were extracted and tabulated. Similarly, the percentage of women receiving the R01-equivalent awards and the success rates by type of application (Type 1/New or Type 2/Renewal) were compared over the study period to examine the temporal trends. The average funding of R01-equivalent grants in current and constant dollars was also compared between genders.

Data analysis

We analyzed the categorical data by gender and its temporal trend by year and across RPGs and R01 awards. The award success rate was compared for RPGs and R01-equivalent grants, and absolute change (%) was calculated from the years 1998 to 2019. Tables were created to highlight and compare the gender percentages for each category and year.

## Results

The contribution and involvement of women in research development from the year 1998 to 2019 were tabulated (Table 1). There has been a substantial increase in the overall percentage of women receiving RPGs at the NIH over the last 21 years and it is continually increasing. There was an absolute increase of 11% from 1998 to 2019 (Table 1).

**Table 1:** Research Project Grants: Awards by Gender and Percentage to Women. (For the last fiscal year displayed, Research Project Grants are defined as activity codes DP1, DP2, DP3, DP4, DP5, P01, PN1, PM1, R00, R01, R03, R15, R21, R22, R23, R29, R33, R34, R35, R36, R37, R61, R50, R55, R56, RC1, RC2, RC3, RC4, RF1, RL1, RL2, RL9, RM1, UA5, UC1, UC2, UC3, UC4, UC7, UF1, UG3, UH2, UH3, UH5, UM1, UM2, U01, U19, and U34. Research projects were first coded to the NLM in the fiscal year 2007. Not all of these activities may be in use by the NIH every year.) NLM, National Library of Medicine; NIH, National Institutes of Health

Year	Women	Men	Percentage to Women
1998	5,983	20,850	22%
1999	6,568	22,261	23%
2000	7,048	23,514	23%
2001	7,586	24,764	23%
2002	8,043	26,082	24%
2003	8,696	27,291	24%
2004	9,091	27,918	25%
2005	9,348	27,595	25%
2006	9,346	27,116	26%
2007	9,519	27,339	26%
2008	9,568	26,582	26%
2009	9,523	25,661	27%
2010	9,564	25,492	27%
2011	9,702	25,113	28%
2012	9,882	24,746	29%
2013	9,514	23,861	29%
2014	9,473	23,208	29%
2015	9,703	23,149	30%
2016	10,150	23,802	30%
2017	10,581	24,210	30%
2018	11,629	25,008	32%
2019	12,539	25,915	33%
Absolute Change (%)			+11%

In the comparison of the competing applications and award success rate, there was a smaller decrease in the observed success rates for female RPG applicants at -9% compared to -12% in their male counterparts (Table 2).

**Table 2:** Research Project Grants: Competing Applications, Awards, and Success Rates, by Gender. (For the last fiscal year displayed, Research Project Grants are defined as activity codes DP1, DP2, DP3, DP4, DP5, P01, PN1, PM1, R00, R01, R03, R15, R21, R22, R23, R29, R33, R34, R35, R36, R37, R61, R50, R55, R56, RC1, RC2, RC3, RC4, RF1, RL1, RL2, RL9, RM1, UA5, UC1, UC2, UC3, UC4, UC7, UF1, UG3, UH2, UH3, UH5, UM1, UM2, U01, U19, and U34. Research projects were first coded to NLM in the fiscal year 2007. Not all of these activities may be in use by the NIH every year.) NLM, National Library of Medicine; NIH, National Institutes of Health

Year	Women			Men		
	Applications	Awards	Success Rate	Applications	Awards	Success Rate
1998	5,763	1,715	30%	17,726	5,698	32%
1999	6,385	1,977	31%	19,114	6,457	34%
2000	6,851	2,046	30%	20,025	6,600	33%
2001	7,188	2,229	31%	20,451	6,754	33%
2002	7,808	2,246	29%	21,699	7,045	32%
2003	9,175	2,733	30%	24,992	7,564	30%
2004	10,873	2,597	24%	29,453	7,406	25%
2005	11,739	2,602	22%	30,696	6,938	23%
2006	12,355	2,452	20%	32,329	6,599	20%
2007	13,131	2,706	21%	32,971	7,294	22%
2008	12,489	2,706	22%	30,271	6,656	22%
2009	12,894	2,546	20%	29,686	6,264	21%
2010	13,956	2,673	19%	31,307	6,703	21%
2011	15,126	2,587	17%	33,657	6,104	18%
2012	15,417	2,695	17%	34,685	6,214	18%
2013	14,969	2,408	16%	32,696	5,742	18%
2014	15,428	2,777	18%	33,477	6,268	19%
2015	15,798	2,891	18%	34,396	6,497	19%
2016	16,789	3,159	19%	35,370	7,028	20%
2017	16,954	3,186	19%	34,930	6,763	19%
2018	17,651	3,687	21%	35,027	7,164	20%
2019	17,857	3,705	21%	34,844	7,086	20%
Absolute Change (%)			-9%	Absolute Change (%)		-12%

Women had a significant increase from 5,203 to 9,263 between 1998 and 2019 as the recipients of R01 grants (Table 3). The percentage of total R01 grants received by women increased by 9% in the same period.

**Table 3:** R01-Equivalent Grants: Awards by Gender and Percentage to Women. (For the last fiscal year displayed, R01-equivalent grants are defined as activity codes DP1, DP2, DP5, R01, R37, R56, RF1, RL1, U01, and R35 from select NIGMS and NHGRI program announcements. Not all of these activities may be in use by the NIH every year.) NIGMS, National Institute of General Medical Sciences; NHGRI, National Human Genome Research Institute; NIH, National Institutes of Health

Year	Women	Men	Percentage to Women
1998	5,203	18,297	22%
1999	5,665	19,471	22%
2000	5,982	20,348	22%
2001	6,269	21,019	22%
2002	6,498	21,638	23%
2003	6,825	22,057	23%
2004	7,025	22,209	23%
2005	7,066	21,841	24%
2006	7,046	21,419	24%
2007	7,042	21,231	25%
2008	6,949	20,628	25%
2009	6,958	20,166	26%
2010	7,108	20,162	26%
2011	7,088	19,496	27%
2012	7,039	18,956	27%
2013	6,714	18,080	27%
2014	6,572	17,212	28%
2015	6,606	16,805	28%
2016	6,877	17,091	29%
2017	7,238	17,353	29%
2018	8,660	19,820	30%
2019	9,263	20,389	31%
Absolute Change (%)			+9%

In a comparison of R01 grant funding in current and constant dollars, the total funding amount for women was greater in 2019, which is an improvement from 1998. In 1998, men received higher funding amounts in both current and constant dollars (Table 4).

**Table 4:** R01-Equivalent Grants: Average Funding in Current and Constant Dollars, by Gender. (Current dollars and constant dollars represent average costs. Constant dollars were computed using 1998 as the base from the BRDPI based on the latest FY. Constant dollar figures were not yet available for FY2019. For the last FY displayed, R01-equivalent grants are defined as activity codes DP1, DP2, DP5, R01, R37, R56, RF1, RL1, U01, and R35 from select NIGMS and NHGRI program announcements. Not all of these activities may be in use by the NIH every year.) BRDPI, Biomedical Research and Development Price Index; FY, fiscal year; NIGMS, National Institute of General Medical Sciences; NHGRI, National Human Genome Research Institute; NIH, National Institutes of Health

Year	Current Dollars		Constant Dollars (1998)	
	Women	Men	Women	Men
1998	$240,912	$247,820	$240,912	$247,820
1999	$261,864	$262,583	$253,744	$254,441
2000	$284,727	$281,903	$266,054	$263,415
2001	$308,613	$302,894	$279,161	$273,988
2002	$329,186	$323,274	$288,259	$283,082
2003	$349,085	$338,870	$295,346	$286,704
2004	$358,540	$350,462	$292,523	$285,932
2005	$367,410	$359,970	$288,508	$282,665
2006	$367,326	$359,554	$275,757	$269,922
2007	$371,142	$360,291	$268,422	$260,574
2008	$384,534	$370,403	$265,623	$255,862
2009	$400,568	$384,358	$268,900	$258,019
2010	$413,027	$395,426	$269,712	$258,218
2011	$417,379	$400,785	$264,873	$254,342
2012	$430,065	$410,383	$275,178	$262,585
2013	$415,576	$398,015	$261,407	$250,360
2014	$442,529	$421,615	$266,229	$253,647
2015	$450,840	$429,555	$265,297	$252,772
2016	$478,624	$450,130	$275,647	$259,237
2017	$505,649	$472,930	$283,828	$265,462
2018	$547,492	$528,776	$299,279	$289,048
2019	$564,094	$541,184	NA	NA

When examining trend by the application type of R01 grants, women had a small decrease in the success rates for both Type 1/New and Type 2/Renewal types of R01 grants, thus showing an overall better success rate in women for Type 1/New R01 grants and in men for Type 2/Renewal R01 grants (Table 5).

**Table 5:** R01-Equivalent Grants: Success Rates, by Gender and Type of Application. (For the last fiscal year displayed, R01-equivalent grants are defined as activity codes DP1, DP2, DP5, R01, R37, R56, RF1, RL1, U01, and R35 from select NIGMS and NHGRI program announcements. Not all of these activities may be in use by the NIH every year.) NIGMS, National Institute of General Medical Sciences; NHGRI, National Human Genome Research Institute; NIH, National Institutes of Health

Year	Women, New (Type 1)	Men, New (Type 1)	Women, Renewal (Type 2)	Men, Renewal (Type 2)
1998	28%	28%	44%	52%
1999	28%	30%	50%	56%
2000	29%	30%	46%	54%
2001	28%	29%	50%	54%
2002	26%	29%	46%	53%
2003	28%	28%	49%	51%
2004	23%	24%	42%	46%
2005	21%	21%	36%	41%
2006	19%	20%	34%	37%
2007	22%	23%	36%	40%
2008	21%	22%	35%	38%
2009	19%	17%	32%	38%
2010	18%	18%	35%	40%
2011	15%	15%	33%	36%
2012	15%	15%	31%	37%
2013	14%	15%	29%	34%
2014	16%	15%	33%	37%
2015	16%	16%	34%	35%
2016	17%	18%	35%	38%
2017	17%	17%	34%	40%
2018	20%	20%	41%	45%
2019	20%	19%	42%	43%
Absolute change (%)	-8%	-9%	-2%	-9%

## Discussion

We studied gender disparity in NIH grant awards and funding from 1998 to 2019. There was an overall increase in the percentage of female awardees for all categories of RPGs from 22% to 33% between the years 1998 and 2019, with an absolute increase of 11%. The number of female RPG applicants increased from 5,763 to 17,857 from 1998 to 2019. However, the success rates for female RPG applicants decreased from 30% to 21%, with an absolute decrease of 9% for the same period. Women have made progress as recipients of various RPGs; however, the percentage of female awardees is not comparable to their representation in the population. The percentage of R01-equivalent grants awarded to women increased by approximately 22% to 31% between 1998 and 2019, with an absolute increase of 9% [18]. The increase in average funding in current and constant dollars for the R01-equivalent grant awarded to women also increased slightly more than their male counterparts. A study revealed that women with an MD degree were awarded larger NIH R01 grants in obstetrics and gynecology than males with an MD degree between 2008 and 2017 [20].

Our study showed a substantial decline between 1998 and 2007 for women receiving R01 award(s). The percentage of women submitting R01-new and R01-equivalent grants did not change through 2001-2003, whereas the percentage of women submitting R01 grant renewal decreased [21]. For the years 2003 to 2007, there was a significant decline in the percentage of women investigators receiving R01 grants (new and renewal) [18,21]. Analysis of the R01 program data showed that funding and success rates for new R01 awards over the past decade have been almost identical between both the genders, but women were less successful at the renewal of R01-equivalent awards [22]. Higher application and success rates have been observed for men having previous experience as NIH grantees than women at similar career points [1]. Although women secured higher numbers of R01 awards than men, men had a greater number of R01 awards than women at all times in their careers [22].

The previous studies exploring the effects of gender on NIH funding suggested contrasting results. A retrospective cohort study (1997-2007) showed that women were less likely than men to receive an R01 grant [23]. Another study analyzing the NIH data regarding contending research and training grants suggested that women were equally or more productive than men in the R01 program, both as first-time applicants and as experienced applicants submitting new applications [18]. However, experienced male researchers were more successful than female researchers for R01 Type 2 (renewal) grant submissions [22]. After controlling for all covariates, male PhDs were significantly more likely than female PhDs to have received at least one R01 award [24]. These findings were also consistent with another study exploring the NIH grant funding in radiology. This study observed a significant gender disparity in mean NIH grants awarded to radiology investigators for 2016-2019 inclusive ($619,807.00 for male PhD investigators compared to $158,486.00 for female PhD investigators) [25].

Analysis of a report (2010-2014) regarding NIH peer reviewers’ critique on R01 grant applications showed that gender bias existed in the peer-review process of R01 grants, particularly for R01 type 2 grants (R01-renewal grants) [26-27]. Although the percentage of women who received the R01 awards has been on the rise for the last 20 years, the probable attrition in the promotion of women to senior positions can discourage women from entering and staying in academia [28]. Research has been a driving force in the advancement of medicine, and more than half of the world’s funding comes from the U.S. [1]. In 2014, the R01 grant accounted for 49% of all NIH extramural funding [2]. The R01 grant is also seen as a turning point in the early career of an academician that can be utilized to promote further granting opportunities [29].

Our study revealed existing gender differences in grant awards, which, in turn, warrants further exploration and intervention. Several factors including a career change and differing grant application strategies may be involved in a high female dropoff rate at first renewal in NIH funding. Further work needs to be conducted to address the explanation why women in academic positions might not be applying or reapplying for RPGs at the same rates as men and how this pattern could be changed [1]. Furthermore, an investigation into why women receive less favorable reviews than men for renewal applications is underway [30]. Over the decade studied, a gender disparity exists in the number of total grants and award dollars that are received by primary investigators for NIH R01 grants. However, a trend has been observed for the increased funding for those women who receive an NIH R01 grant. To combat these gender disparities, further research needs to be conducted to determine the cause and implement remedial actions [29].

There are limitations to our study. In the context of gender disparity, some researchers may self-identify in a non-binary fashion. Furthermore, the data on NIH database are only comprised of information related to those who receive funding. There are no data available on the total number of applications, including male and female applicants. Therefore, the true determination of the relative success rate of male and female researchers and differences in award amounts is not possible. Also, each application for NIH funding includes various personal information such as the name, degree, position, and academic title of the researcher, among others. It is, therefore, plausible that funding decisions are made with the knowledge of an applicant’s gender, although the extent to which this knowledge may affect funding decisions, whether explicitly or through implicit bias, is impossible to quantify objectively.

## Conclusions

Women have made progress as recipients of various RPGs. However, the percentage of female awardees is not comparable to the higher numbers of female doctoral candidates in the U.S. Despite an overall increase in the percentage of female researchers successfully receiving NIH grants and awards, the gender disparity exists. As apparent from the increasing enrollment of women in medical schools as well as doctoral candidates, and considering the lengthy training periods, it will be a few years before we can see meaningful changes in bridging gender disparity. Therefore, further studies to examine the longitudinal trends and lag times of women dissipating the gender differences are needed. At the same time, the continued support and retention of female researchers are pivotal to further improve the future representation of women in research, and specifically at the NIH.
